# Climatic and biogeographic processes underlying the diversification of the pantropical flowering plant family Annonaceae

**DOI:** 10.3389/fpls.2024.1287171

**Published:** 2024-03-08

**Authors:** Weixi Li, Runxi Wang, Ming-Fai Liu, Ryan A. Folk, Bine Xue, Richard M. K. Saunders

**Affiliations:** ^1^ Division of Ecology & Biodiversity, School of Biological Sciences, The University of Hong Kong, Hong Kong, Hong Kong SAR, China; ^2^ Department of Biological Sciences, Mississippi State University, Starkville, MS, United States; ^3^ College of Horticulture and Landscape Architecture, Zhongkai University of Agriculture and Engineering, Guangzhou, China

**Keywords:** diversification rate shift, global temperature, biogeographical events, climatic niche evolution, diversification process

## Abstract

Tropical forests harbor the richest biodiversity among terrestrial ecosystems, but few studies have addressed the underlying processes of species diversification in these ecosystems. We use the pantropical flowering plant family Annonaceae as a study system to investigate how climate and biogeographic events contribute to diversification. A super-matrix phylogeny comprising 835 taxa (34% of Annonaceae species) based on eight chloroplast regions was used in this study. We show that global temperature may better explain the recent rapid diversification in Annonaceae than time and constant models. Accelerated accumulation of niche divergence (around 15 Ma) lags behind the increase of diversification rate (around 25 Ma), reflecting a heterogeneous transition to recent diversity increases. Biogeographic events are related to only two of the five diversification rate shifts detected. Shifts in niche evolution nevertheless appear to be associated with increasingly seasonal environments. Our results do not support the direct correlation of any particular climatic niche shifts or historical biogeographical event with shifts in diversification rate. Instead, we suggest that Annonaceae diversification can lead to later niche divergence as a result of increasing interspecific competition arising from species accumulation. Shifts in niche evolution appear to be associated with increasingly seasonal environments. Our results highlight the complexity of diversification in taxa with long evolutionary histories.

## 1 Introduction

Tropical forests occupy almost 8 million km^2^ of the humid tropics and greatly contribute to the species diversity of forest ecosystems (Myers, 1991; Lewis, 2006; Wright, 2010). Tropical forest biomes face the threat of extinction associated with climate change (Laurance, 2013; Deb et al., 2018), which has significantly influenced the distribution and biological properties of vegetation, and hence shaped plant diversity (Watson et al., 1996; Huntley, 1997; Bakkenes et al., 2002; Thuiller et al., 2005; Becklin et al., 2016). The processes and mechanisms underlying species diversification in tropical forests remain unclear, hampering the assessment of how these ecosystems respond to global climate change, and hence impeding the development of conservation priorities in the face of future changes.

Diversification is shaped by three key processes: speciation, extinction, and migration, which are the raw materials upon which climate change and other factors operate to cause changes in local or global biodiversity (Wüest et al., 2019; Cai et al., 2020). Current evidence suggests that migration of plant lineages in tropical forests is very limited (Donoghue and Edwards, 2014), however, implying that speciation and extinction are perhaps key to species diversification of forests. The link between speciation, extinction and the modern-day diversity is influenced by age and rates: in other words, the relationship between the very high species richness of forests and evolutionary rates in these biomes is not straightforward.

Multiple factors have been proposed as drivers of diversification through the above three processes. On a geological timescale, global climate changes have repeatedly been shown to significantly influence diversification (Erwin, 2009). Recent documented botanical examples include *Primulina* (Gesneriaceae; Kong et al., 2017), Saxifragales (Folk et al., 2019), Rosids (Sun et al., 2020), Asteraceae (Zhang et al., 2021), *Scleria* (Cyperaceae; Larridon et al., 2021), and *Oreocharis* (Gesneriaceae; Kong et al., 2022). Global temperature has fluctuated dramatically throughout Earth’s history (Zachos et al., 2001; Hansen et al., 2006; Rahmstorf et al., 2017) and is considered to play a crucial role in shaping the fate of clades and the biomes that they are adapted to. Two contrasting evolutionary mechanisms might be invoked to explain how a taxon responds to global temperature variation: “key innovations” involving intrinsic biotic factors, such as morphological or behavioral changes, can enable lineages to overcome abiotic stressors and facilitate novel niche expansions (Moore and Donoghue, 2007; Larridon et al., 2021). By contrast, “key opportunities” represent instances in which a lineage migrates to a new region (Moore and Donoghue, 2007). Key opportunities may be linked to the development of novel adaptations, such as key innovations, facilitating exploration of new environments. The adaptation of organisms to new climatic conditions, reflected in niche evolution, suggests a connection to key opportunities. Numerous studies suggest key innovations and key opportunities can play a synergistic role in species diversification (Moore and Donoghue, 2007; Larridon et al., 2021). Integrating temperature- and state-dependent diversification models and conducting comparative analyses would help disentangle the association between key opportunities, key innovations and tropical diversity. Furthermore, it would help distinguish among hypotheses explaining the distribution of this diversity.

The family Annonaceae (Magnoliales) represents a study model for understanding the macroevolution of tropical forests. The family originated in the early Cretaceous (112.6–89 Ma; Xue et al., 2020). It is a large and highly diverse pantropical lineage, comprising approximately 2440 species, widely distributed in tropical lowland forests (Couvreur et al., 2012). The distribution pattern of Annonaceae closely corresponds to the tropical climate, with only limited representation in frost-prone areas (Couvreur et al., 2011), implying that the climatic niche and historical biogeography of Annonaceae may be closely linked to the expansion and contraction of tropical forests. A previous study (Couvreur et al., 2011) has suggested that lineages within Annonaceae accumulated through stable diversification rates over time, although the limited taxon sampling (4.8% of species) is likely to have resulted in an underestimation of diversification rate. More recent research (Xue et al., 2020) with improved sampling (34.2% of species) proposes an accelerated diversification in Annonaceae around 25 Ma. Certain biotic factors in Annonaceae, such as pollinator trapping, androdioecy, and single-seeded monocarp fragments, are possibly correlated with high diversification rates (Xue et al., 2020). The contribution of biogeography and climate change to the rapid diversification rate increase has yet to be investigated, however. The combination of incomplete sampling in previous work on the family (Couvreur et al., 2011), uncertainty about the timing and drivers of diversification, and the paucity of studies concerning the evolution of pantropical forests suggests that our overall framework for understanding tropical diversification is incomplete. There is therefore a need to use an enriched phylogenetic tree to explore the macroevolution of Annonaceae and enhance our understanding of tropical forest diversification.

Correlations between key opportunities or key innovations and diversification rate shifts in other plant families are not always deterministic (Moore and Donoghue, 2007; Larridon et al., 2021; Stull et al., 2021), highlighting the need to consider multiple factors. Key opportunities here represent lineages migrating to a new region that provides a similar environment (Moore and Donoghue, 2007), and key innovations here refer to the climatic niche resulting from innovations in intrinsic traits. Other localized opportunities, such as changing climates *in situ* following the uplift of mountains, and morphological trait innovations explored by Xue et al. (2020), are not considered in this study. In a recent study by Wang et al. (2021), a correlation was revealed between the rate of climate niche evolution and high diversification rate in Annonaceae. However, the specific sequence and interplay between key innovation and key opportunities in relation to diversification remain obscure and require further investigation to fully understand tropical plant diversification models. This study aims to explore the following hypotheses: (1) Global temperature change may have driven the recent acceleration in diversification of Annonaceae; (2) Niche divergence may promote the recent acceleration of diversification; and (3) Biogeographical events may also have driven the diversification of Annonaceae, possibly due to their role in promoting niche evolution.

## 2 Materials and methods

### 2.1 Data sampling

Maximum likelihood (ML) analysis results were obtained from Xue et al. (2020) with 1000 rapid bootstrapping of 923 species. The dataset comprises 916 Annonaceae ingroup and seven outgroup taxa following concatenation of eight chloroplast genes or gene spacer regions. A pruned tree file containing 835 tips was generated after excluding outgroup taxa and the removal of undescribed and unidentified species to eliminate potential misidentifications or uncertainties.

### 2.2 Phylogenetic inference and diversification analyses

Divergence times were estimated using the penalized likelihood method implemented in the treePL software (Smith and O’Meara, 2012) due to its efficiency in handling large datasets compared to other tools such as BEAST. The input trees were all bootstrapped ML trees from Xue et al. (2020) after pruning taxa as described in “Data sampling” with branch lengths preserved. The root age of the phylogenetic tree was fixed at 137 Ma based on the mean divergence time between Magnoliales and Laurales (Foster et al., 2017), which was also supported by the occurrence of a pollen grain fossil record representing an unequivocal crown group angiosperm (Early Cretaceous, 136.4–130 Ma: Hughes, 1994; Friis et al., 2010). For other calibrations, the Annonaceae crown node was set between 112.6 and 89 Ma, while the Magnoliineae crown node was placed between 125 and 112.6 Ma (Thomas et al., 2015, 2017; Xue et al., 2020). We furthermore conducted an additional dating analysis based on the best ML tree with the root age calibrated between 159.91–120.59 Ma, considering a 95% confidence interval (CI) between Magnoliales and Laurales, as recovered by Foster et al. (2017); other calibrations were set as above.

### 2.3 Paleotemperature-dependent diversification

We performed a global temperature-dependent diversification analysis using the 835-taxon tree. The mean global Cenozoic paleotemperature curve, derived from carbon isotopes (Zachos et al., 2008), served as the basis for our analysis. Two constant rate models and eight time-dependent birth-death models were furthermore included in the model set for comparison. The constant rate models were: (1) BCST: pure birth model with constant speciation rate with no extinction; and (2) BCST DCST: both speciation and extinction rates are constant. The eight time-dependent birth-death models were: (3) BTimeVar_EXPO: speciation rates vary exponentially through time with no extinction; (4) BTimeVarDCST_EXPO: speciation rates vary exponentially through time, while the extinction rate remains constant; (5) BCSTDTimeVar_EXPO: extinction rates vary exponentially through time, while the speciation rate is constant; (6) BTimeVarDTimeVar_EXPO: both speciation and extinction rates vary exponentially through time; (7) BTimeVar_LIN: speciation rates vary linearly through time with no extinction; (8) BTimeVarDCST_LIN: speciation rates vary linearly through time, while the extinction rate is constant; (9) BCSTDTimeVar_LIN: extinction rates vary linearly through time, while the speciation rate is constant; and (10) BTimeVarDTimeVar_LIN: both speciation and extinction rates vary linearly through time. The “fit_env function” in the R-package RPANDA v.1.1 (Morlon et al., 2016) was employed to evaluate the best diversification model. Eight models were fitted to environmental data, as follows: (11) BTempVar EXPO: speciation rates vary exponentially with environmental data, while the extinction rate is fixed at zero (unparameterized); (12) BTempVar DCST EXPO: speciation rates vary exponentially with environmental data, while the extinction rate remains constant; (13) BCSTDTempVar EXPO: extinction rates vary exponentially with environmental data, while the speciation rate is constant; (14) BTempVar DTempVar EXPO: both speciation and extinction rates vary exponentially with environmental data; (15) BTempVar_LIN: speciation rates vary linearly with environmental data, while the extinction rate is fixed at zero; (16) BTempVarDCST_LIN: speciation rates vary linearly with environmental data, while the extinction rate is constant; (17) BCSTDTempVar LIN: extinction rates vary linearly with environmental data, while the speciation rate is constant; and (18) BTempVar DTempVar_LIN: both speciation and extinction rates vary linearly with environmental data. Comparison of these models enables the identification of the best-fitting model that describes the relationship between temperature and diversification rates. The results were also compared to RevBayes (Höhna et al., 2016) estimations based on episodic models: (1) a model with constant birth and death rate within an interval but allowing for instantaneous shifts to new rates during a rate-shift episode (May et al., 2016; Magee et al., 2020); and (2) an environmental-dependent model to test for a correlation between diversification rate and global paleotemperature.

### 2.4 Biogeography

The distribution data for the study were obtained from the Global Biodiversity Information Facility (GBIF, 2021; http://www.gbif.org), with additional locality data sourced from iDigBio (https://www.idigbio.org/portal/search). A total of 110,744 records were manually checked and cleaned using “CoordinateCleaner” (Zizka et al., 2019) in R, with 43888 records left for 728 species with specific locations. Species without specific location data were therefore pruned from the 835-taxon tree resulting a tree of 728 species for biogeographical analysis. The same dataset was utilized in the subsequent analyses of climatic niche evolution and clade-dependent diversification to ensure consistency across elements and make the results comparable.

To infer the ancestral areas of extant species and reconstruct dispersal history, all extant species were assigned to six geographical regions based on paleogeographical reconstructions (Couvreur et al., 2011) and current Annonaceae distribution data: (A) Southeast Asia, west of Huxley’s Line; (B) Southeast Asia, east of Huxley’s Line, northern Australia, and Pacific islands; (C) Continental Africa; (D) Madagascar; (E) North/Central America; and (F) South America. The maximum size of reconstructed ancestral areas was set to six regions. Ancestral area reconstruction was performed using BioGeoBears (Matzke, 2013) under four models: Dispersal-extinction-cladogenesis (DEC), and Dispersal Vicariance Analysis (DIVALIKE), and their +j variant (the jump dispersal parameter, allowing for “founder-event speciation”). The best-fitting model was determined based on the lowest corrected Akaike’s information criterion (AICc) values, enabling identification of the scenario that provides the best trade-off between model fit and complexity. Despite DEC+j being identified as the optimal model for this dataset, we used both DEC and DEC+j for ancestral area reconstruction analysis due to concerns about potential statistical issues associated with the DEC+j model (Cornuault and Sanmartín, 2022).

Biogeographical stochastic mapping (BSM) was conducted to estimate the number and type of geodispersal events based on the best-fitting model. The frequencies and directions of these events were determined by calculating the mean and standard deviation (SD) from 50 BSMs. We also estimated the number of dispersal events per 1 million years in BioGeoBears. This analysis allows us to estimate the timing, direction, location, and quantity of biogeographic events. Subsequently, we compared these events with the occurrences of diversification rate shifts and niche evolution shifts to determine if they coincided.

### 2.5 Climatic niche evolution

Bioclimatic data representing 19 temperature and precipitation measures were obtained from Worldclim (https://www.worldclim.org/) to investigate the evolution of climatic preferences of species and their potential association with accelerated diversification in Annonaceae. Mean values for each variable were calculated for every species, using the 728-species dataset used in the biogeography analysis with valid bioclimatic data. Principal Component Analysis (PCA) was employed by using the function “corrplot” (Wei et al., 2017) in R to characterize the variation in climatic variables among species, thereby summarizing variance in the 19 variables into two-dimensional axes for downstream analysis. Mean PC scores were calculated for each species on each axis, providing a representation of their climatic niche. We first used the “fitContinuous” function in R package “geiger” (Pennell et al., 2014) to compare whether a Brownian motion (BM) (Felsenstein, 1973), Ornstein-Uhlenbeck (OU) (Butler and King, 2004) or Early-burst (EB) (Harmon et al., 2010) model fitted the data best, with the best fitting model (OU) used to infer climatic niche diversification rate shifts in the R package “bayou” (Uyeda and Harmon, 2014); this package utilizes a reversible-jump Bayesian method to fit the OU model. rjMCMC (reversible-jump Markov Chain Monte Carlo) sampling was run for 10^6^ cycles to achieve convergence, discarding the first 30% of samples as burn-in. Only one shift per branch was allowed, and shifts with posterior probabilities (PP) above 0.5 were plotted to identify significant shifts in niche lability. We mapped niche shifts onto the phylogenetic tree to explore their correlation with biogeographical events and diversification shifts. Additionally, we aimed to estimate the future direction of niche evolution.

### 2.6 Clade-dependent diversification

Diversification rates through time across the Annonaceae phylogeny based on the 728-taxon tree were estimated using the BAMM v.2.5 (Rabosky et al., 2014). BAMM uses a reversible-jump Markov chain Monte Carlo (rjMCMC) algorithm to explore the parameter space and estimate diversification rates under optimized “configuration” (sets of reconstructed shifts between diversification regimes with parameterizations within each). The MCMC analysis was run for 20 million generations, with sampling every 1,000 generations. After discarding 10% of the output as burn-in, convergence was assessed by reference to effective sample size (ESS) values that exceed 200 for the likelihood. The numbers of shifts were inferred by using the “coda” package v.0.19-1 (Plummer et al., 2006) in R, to ensure adequate sampling from the posterior distribution. After the MCMC analysis, BAMMtools v.2.5 (Rabosky et al., 2014) was utilized to calculate the posterior distribution and to perform downstream analyses. We estimated variations in the net diversification rate over time and identified occurrence of diversification shifts.

### 2.7 SSE analysis

The GeoSSE (Geographic State Speciation and Extinction) model (Goldberg et al., 2011) was employed to investigate the influence of geographical factors on the diversification of Annonaceae based on the 728-species dataset. This model, implemented in the “diversitree” R package (FitzJohn, 2012), allows for the examination of geographic range effects on diversification. Four different scenarios were considered: (1) GeoSSE: range-dependent diversification without hidden state; (2) GeoHiSSE: range-dependent diversification with hidden state; (3) CID GeoSSE: the range-independent diversification without hidden trait; and (4) CID GeoHiSSE: the range-independent diversification with one hidden trait. The model set therefore jointly tests for a diversification effect and for hidden states (diversification unexplained by measured states). We applied these model comparison analyses to the whole tree based on the results from two clades where diversification shifts and vicariance events were mapped: (1) for one scenario, South America was treated as state 1, and all other areas combined were treated as state 2; and (2) for the other scenario, Africa was treated as state 1, and all other areas combined were treated as state 2. This binary treatment allowed for the evaluation of how geographic range influences the diversification dynamics of Annonaceae, and allowed the detection of any unobserved characters that might impact diversification rates by testing the best-fitting model.

Quantitative analyses were conducted using the QuaSSE (Quantitative State Speciation and Extinction) model, implemented in the “diversitree” R package (FitzJohn, 2012), to examine the interplay between niche lability and diversification rates based on the 728-species dataset. Two principal component (PC) axes derived from 19 climatic variables were selected to estimate the evolution of speciation and extinction rates. The QuaSSE model incorporates a birth-death process, in which speciation (λ) and extinction rates (μ) are determined by a quantitative trait. We fitted three sets of QuaSSE models to explore different relationships between niche lability and speciation: (1) independent speciation rate; (2) speciation that varies as a linear function of PC values; (3) speciation that varies as a sigmoidal function of PC values; and (4) speciation that varies as a hump function of PC values. The models were fitted both with and without the directional component (φ) to capture potential directional evolution of the trait (FitzJohn, 2010). By conducting QuaSSE model testing, we can understand the relationship between the increase or decrease in speciation rate and PC values. For all model comparisons, Akaike information criterion (AIC) values were used to determine the best-fitting models.

## 3 Results

### 3.1 Paleotemperature-dependent diversification

**Figure 1:**
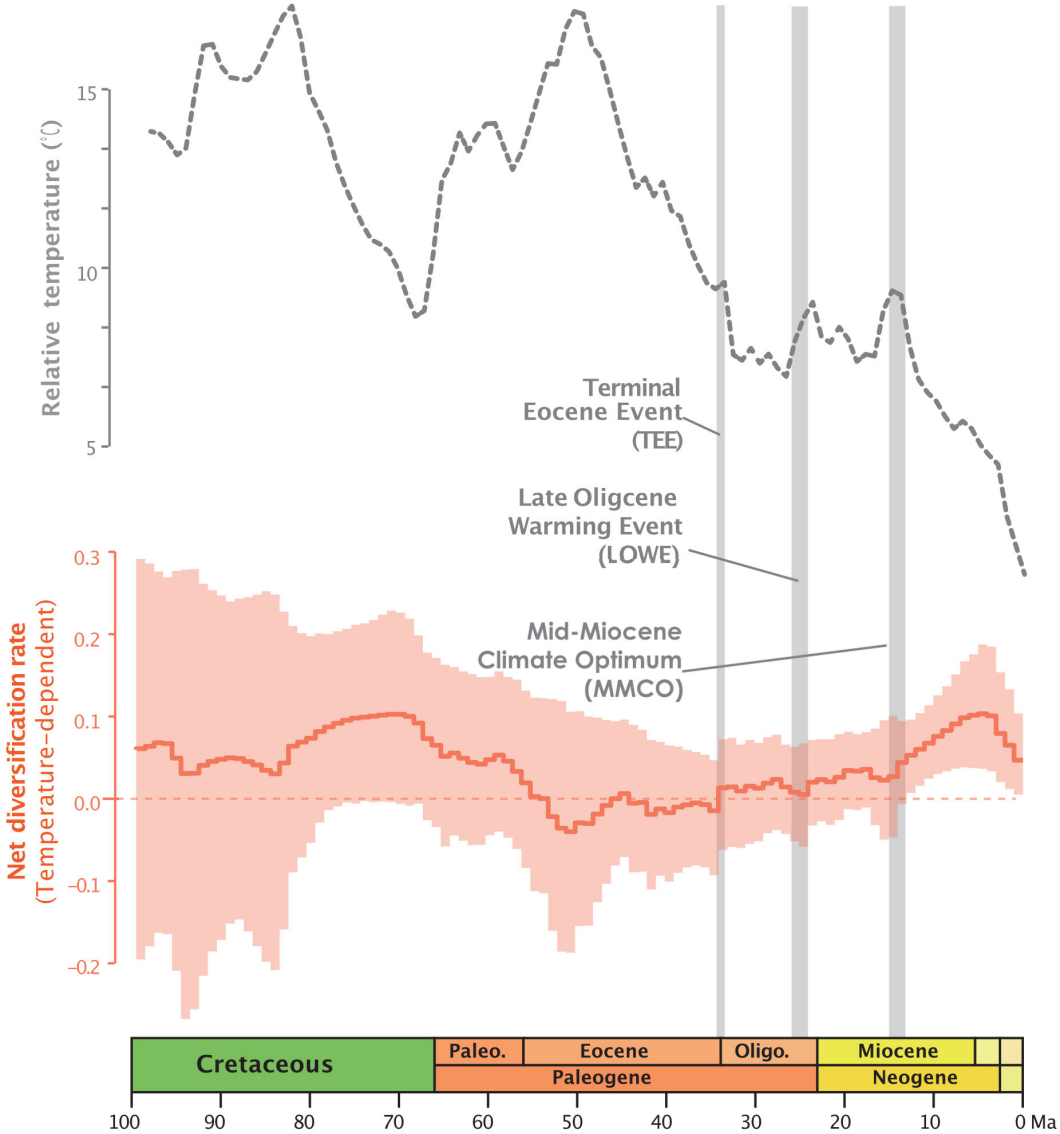
Global relative paleotemperature plot (gray dotted line) and net diversification rate through time plot as a function of temperature from RevBayes analysis for Annonaceae (orange plot: light orange area representing the credibility interval, and the continuous line representing the median rate).

In the RPANDA analysis (**Supplementary Table S1**), the best-supported model with the lowest AICc value (5128.185, AICcω=1) indicates that both speciation and extinction rates are significantly positively correlated with paleotemperature, with positive alpha and positive beta values, respectively. The result of paleotemperature-dependent diversification based on RevBayes analysis, also indicates that both speciation and extinction rates are positively correlated with paleotemperature (**Supplementary Figures S1E, F**). The temperature-dependent net diversification rates plot, represented by the orange line in **Figure 1**, demonstrates that the net diversification rate of Annonaceae has significantly exceeded zero since ca. 25 Ma. The rapid increase from the late Oligocene has been observed in both time and temperature-dependent net diversification rates (**Figure 1**; **Supplementary Figures S1C, G**). The episodic model results obtained from RevBayes demonstrate speciation rates over time that resemble the net diversification rate curve, while the extinction rate remains relatively constant over time (**Supplementary Figures S1A, B**).

### 3.2 Clade-dependent diversification

**Figure 2:**
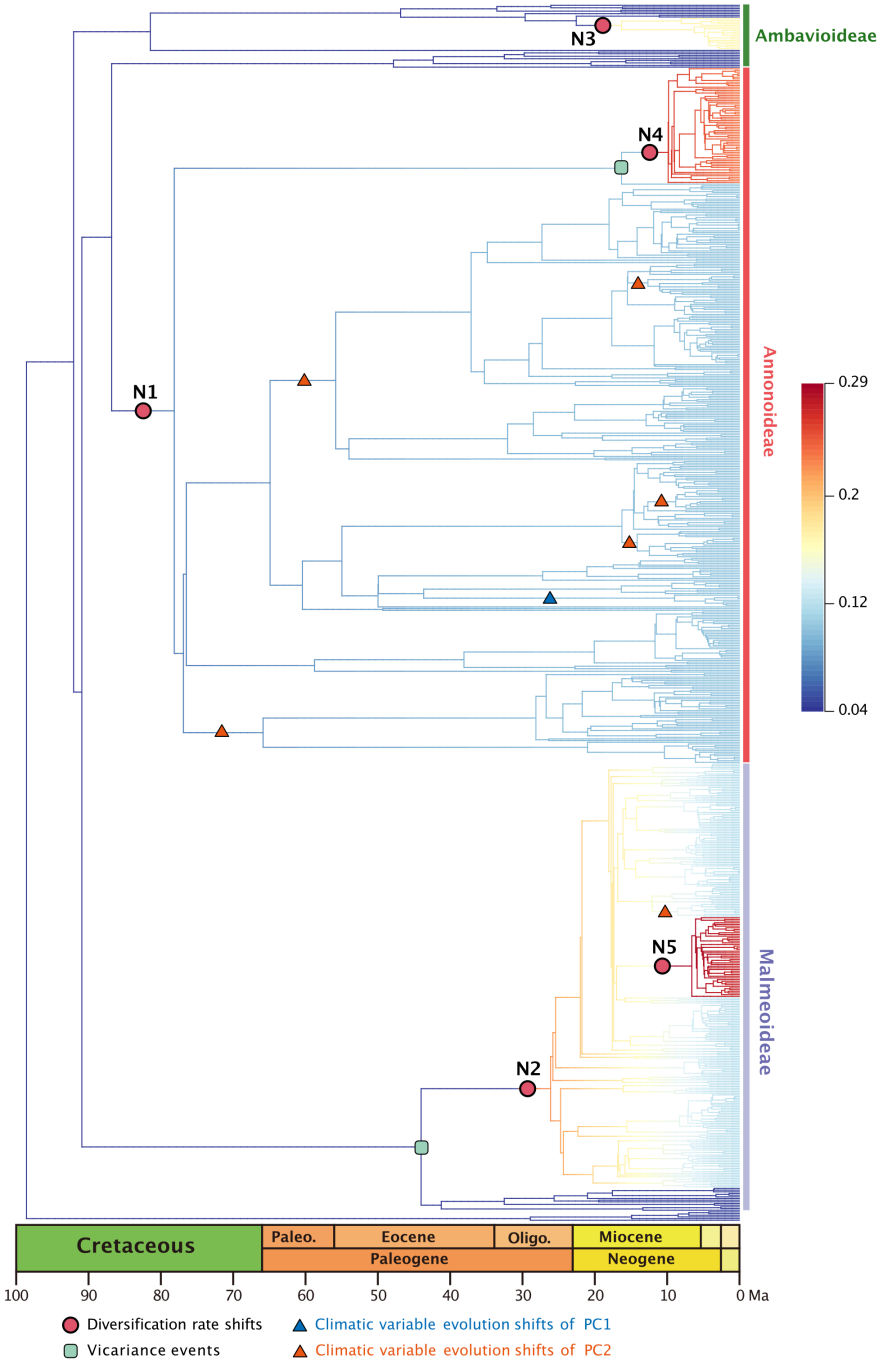
Best BAMM scenario, with diversification rate shifts (shown as red dots) in Annonaceae, mapped positions of vicariance events (shown in the green square), climatic variable evolution shifts of PC1 (shown as blue triangle), climatic variable evolution shifts of PC2 (shown as red triangle). Branch colors indicating diversification rates (refer to the **Supplementary Figure S5** for higher resolution).

The best diversification rate shifts configuration estimation based on the 728-taxon tree reveals rate shifts in five clades (**Figure 2**). Not all nodes in this 728-taxon phylogenetic tree have been well resolved, however: 50.8% (369/727) of nodes exhibit a maximum likelihood bootstrap support > 70%, indicating that half of the branch relationships in this dataset have been fully resolved; 14.0% (102/727) of nodes have a bootstrap support between 50%–70%, suggesting weak support; the remaining 35.2% (256/727) of nodes have a bootstrap support < 50%, indicating that one third of the branch relationships remain unresolved (**Supplementary Figure S2**). The results from two root calibration dating schemes, one fixed at 137 Ma and the other set between 159.91–120.59 Ma, closely resemble each other (**Supplementary Table S2**; **Supplementary Figure S3**).

The earliest rate shift occurred at the branch with the largest number of tribes within subfamily Annonoideae (excluding tribe Bocageeae) (**Figure 2**: N1). This was followed by four subsequent rate shifts: in subfamily Malmeoideae (excluding tribe Piptostigmateae) (**Figure 2**: N2); basal to the *Cyathocalyx*-*Drepananthus* clade within subfamily Ambavioideae (**Figure 2**: N3); in *Guatteria* (subfamily Annonoideae tribe Guatterieae, excluding *Guatteria anomala*) (**Figure 2**: N4); and basal to *Pseuduvaria* (subfamily Malmeoideae tribe Miliuseae) (**Figure 2**: N5).

### 3.3 Biogeographical events

**Figure 3:**
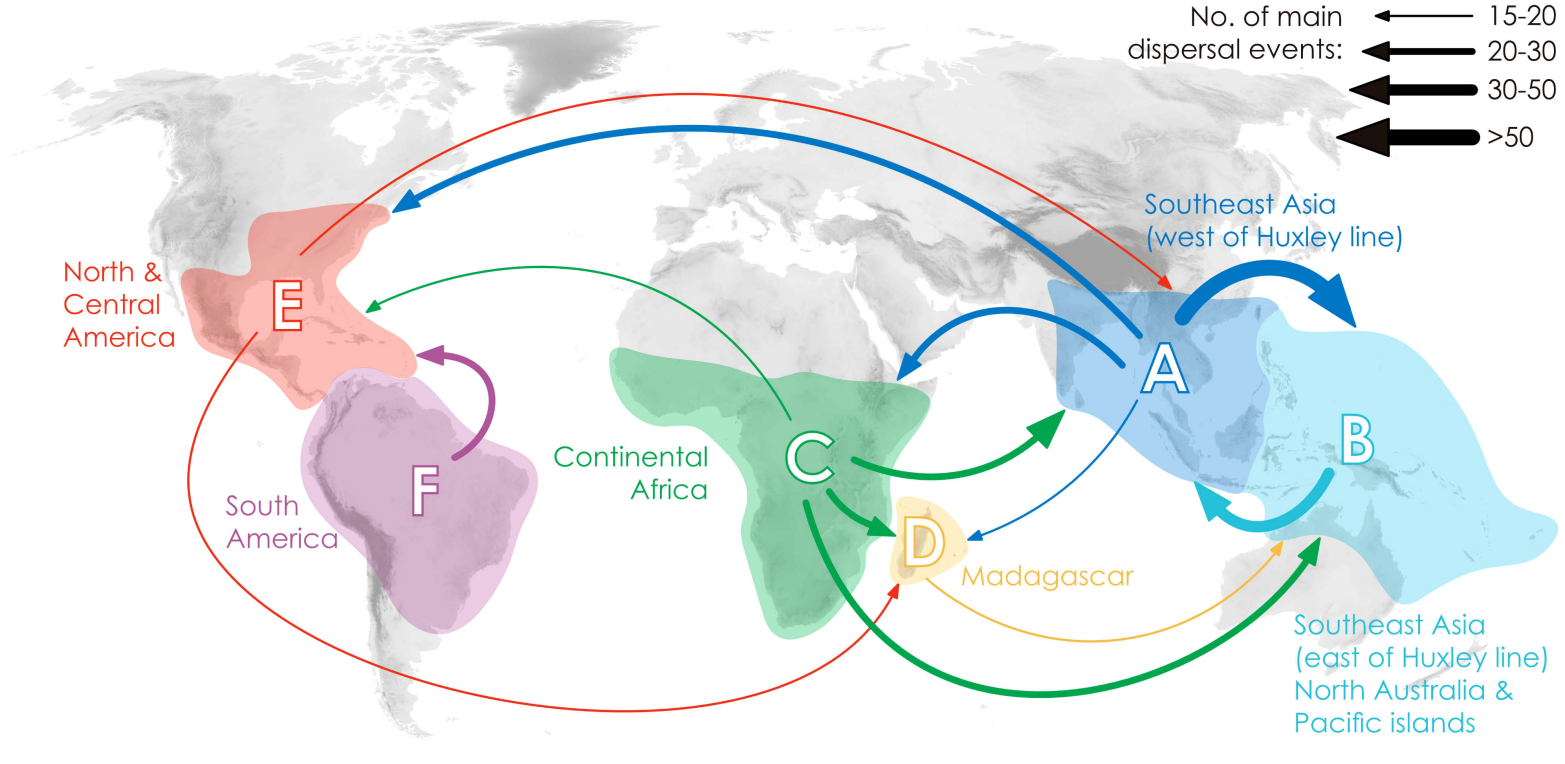
Delimitation of six geographical areas, and dispersal rate between different geographical areas (the thickness of the arrows represents the transition rates and transition directions).

Annonaceae are widely distributed across all six biogeographic regions (**Figure 3**; **Supplementary Figure S4**). The best-fitting model for ancestral area reconstruction was DEC+j based on the 728-taxon tree (**Supplementary Table S3**). BSM analysis identified 1191 biogeographic events (**Supplementary Table S4D**), with cladogenetic events 22% more frequent than anagenetic events. *In situ* speciation within an area accounted for 53.3% of events, vicariance for 4%, and dispersal for 42.5%, including cladogenetic (3.7%) and anagenetic (38.8%) dispersals (**Supplementary Table S4D**). Areas B, C and F (eastern Southeast Asia, continental Africa, and South America) were common sources of dispersal events (**Supplementary Table S4**). BSM analysis identified significant long-distance dispersal (LDD) events (**Figure 3**), with prominent asymmetric floristic exchange observed (**Supplementary Table S4A**): the magnitude between A and B (western and eastern Southeast Asia) is 2.4:1 (A to B: B to A), and between E and F (North/Central and South America) is 2.7:1 (F to E: E to F). Under the DEC+j model, a total of 119 dispersal events and 32 vicariance events were recorded in 425 Annonoideae species, while 61 dispersal events and 19 vicariance events were observed in 266 Malmeoideae species (**Supplementary Figure S4**). Two of the four recent diversification rate shifts were recovered on the same node as reconstructed biogeographical events (**Figure 2**): one was a vicariance event between *Guatteria* and its sister clade, while the other was a vicariance event in subfamily Malmeoideae, between tribe Bocageeae and the remaining tribes. The former vicariance event is inferred to have taken place between South America and North America, resulting in rapid diversification in the South American clade (**Figure 2**). GeoSSE model tests reveal that the best-fitting model was the CID GeoSSE model with no range-dependent diversification, having the lowest AIC value (5115.024, AICω = 0.93) (**Supplementary Table S5**). This suggests that the diversification rate for lineages is unrelated to endemicity in South America (**Supplementary Table S5**). Consistent with this, other clades in South America do not exhibit fast diversification rate shifts. Vicariance in the Malmeoideae was inferred to have occurred between Africa and other areas combined, suggesting that the clade underwent rapid diversification out of Africa after the event (**Figure 2**). The GeoSSE model test showed that the best-fitting model was the CID GeoHiSSE model with one hidden trait but without range-dependent diversification, having the lowest AIC value (5252.59, AICω = 1) (**Supplementary Table S6**), indicating diversification rate for lineages is unrelated to areas.

In general, the ancestral area reconstruction results from the DEC model (**Supplementary Figure S7**) closely align with those of DEC+j (**Supplementary Figure S4**). However, a distinct scenario is evident in the vicariance event between South America and North America (**Figure 2**: N4) when comparing the DEC and DEC+j models.

### 3.4 Climatic niche evolution

**Figure 4:**
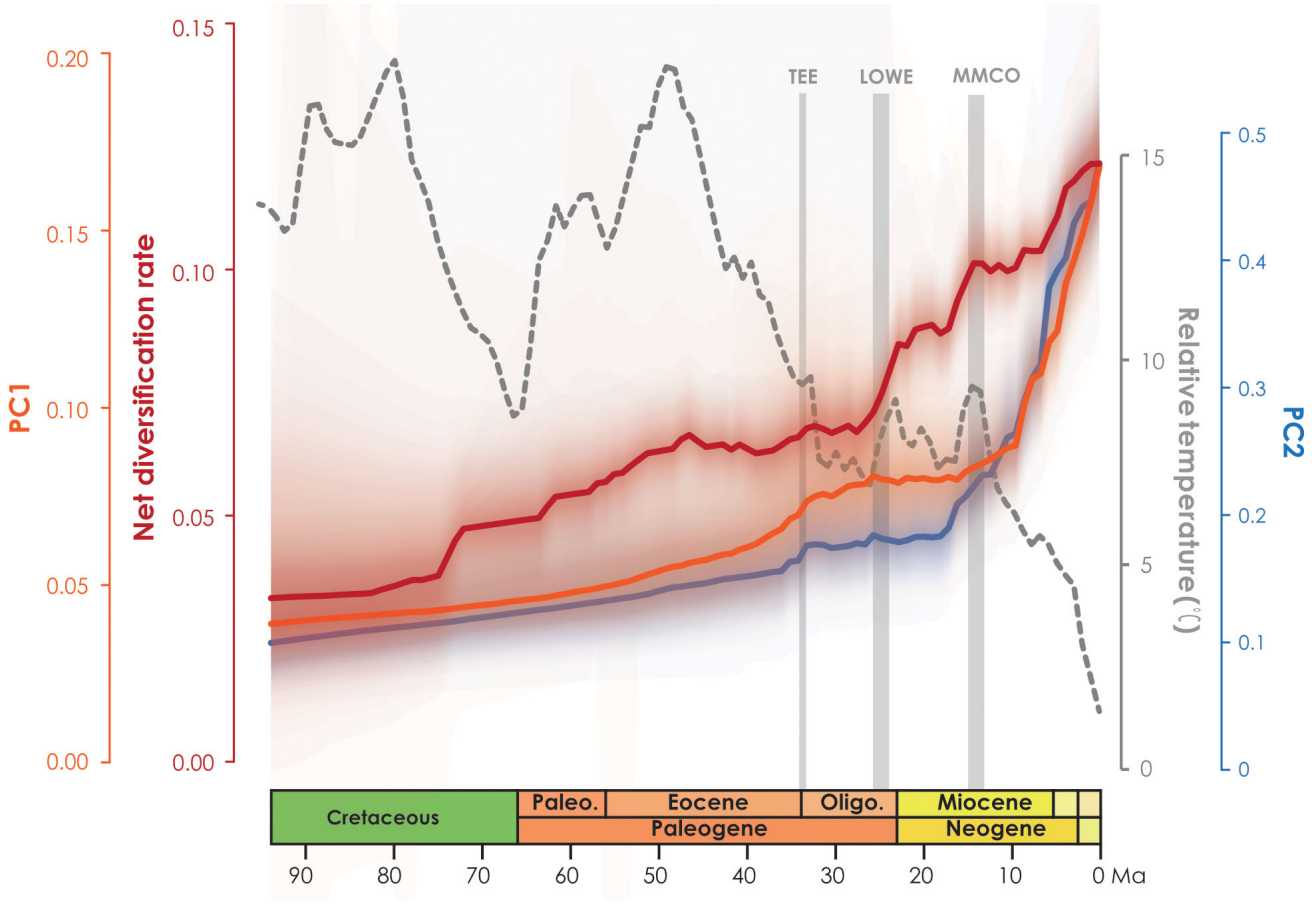
Net diversification rate based on BAMM analysis (red line) and climatic PC1 and PC2 evolution rates over time (orange line and blue line, respectively).

In the PCA analysis based on the 728-species dataset, PC1 primarily captures the annual stability and the minimum extreme temperature during the driest and coldest period, whereas PC2 reflects precipitation during the driest period (**Supplementary Table S7**). The OU model was assumed to be the best model tested based on its lowest AICc score (1997.466 with AICcω = 1 for PC1, and 2791.795 with AICcω = 1 for PC2) (**Supplementary Tables S8**, **S9**). Under the OU model, a significant evolutionary niche shift (PP = 1) was identified at the *Asimina* crown node (**Figure 2**; **Supplementary Figure S6**) within PC1. In PC2, a total of six optimal evolutionary shifts (PP > 0.5) were observed, with five shifts occurring in subfamily Annonoideae and one in subfamily Malmeoideae (**Figure 2**; **Supplementary Figure S6**). No biogeographical events (as determined by DEC and DEC+j) or species diversification rate shifts were directly associated with these niche evolutionary shifts (**Figure 2**; **Supplementary Figure S7**). The timing plot (**Figure 4**) notably displayed a rapid increase in species diversification rates around 25 Ma, followed by a sudden divergence in climatic niche values around 15 Ma, further suggesting a difference in timing between these two evolutionary rates.

QuaSSE analyses suggest the following: PC1 fits more with a drift sigmoidal model (AIC = 6505.917, AICc ω = 0.998) with a negative trend (drift = -0.022), indicating that species with higher PC1 values had lower speciation rates; while PC2 fits more with a drift hump model (AIC = 7387.070, AICc ω = 0.897) with a positive trend (drift = 0.008) (**Supplementary Tables S10**, **S11**), which indicates species with the highest and lowest PC2 values had lower speciation rates. The QuaSSE result supports the general tendency that species with higher PC values have lower speciation rates, indicating increase in temperature extremes (cooler and drier) is associated with speciation rates across clades.

Climatic niche reconstruction (**Supplementary Figure S6**) shows that most of the jumps with higher PP values in the reconstruction results of PC1 are associated with lower values (yellow to red), indicating that jumps in Annonaceae tend to be in response to increased climatic extremes (cooler and drier climate). In the ancestral reconstruction results of PC2, the jumps with larger PP values are likewise all towards lower values (yellow to orange), indicating that the clade of these jumps are in response to increasingly arid climatic extremes.

## 4 Discussion

### 4.1 Does global temperature drive diversification in Annonaceae?

Temperature-dependent analyses suggest that global temperature change may better explain diversification of Annonaceae than time and constant models. RPANDA model tests furthermore indicate that a model where diversification is a function of paleotemperature changes fits the data better than a model in which diversification is independent of changes in paleotemperature. The rapid increase from ca. 25 Ma reported by Xue et al. (2020) aligns with the trends observed in both time and temperature-dependent net diversification rates (**Figure 1**; **Supplementary Figures S1C, G**). Our results support the hypothesis that paleotemperature may have an impact on diversification of Annonaceae, and this paleotemperature-dependent diversification has been documented in both tropical and non-tropical plant lineages, such as *Oreocharis* (Gesneriaceae; Kong et al., 2022), *Primula* (Primulaceae; Smyčka et al., 2022), and rosids (Sun et al., 2020).

Ramirez-Barahona et al. (2020) have observed an obvious delay, averaging 37-56 Ma, between the origin of families (stem age) and the diversification of extant species (crown age) in angiosperms. This observation is consistent with our analyses of Annonaceae. The five identified diversification rate shifts in our study (**Figure 2**) exhibit slight variations compared to those reported by Xue et al. (2020), possibly due to the exclusion of taxa without specific locality records in our analysis. However, not all diversification rate shifts can be directly attributed to global climate events, suggesting the involvement of other factors in macroevolutionary processes. In terms of specific clades within Annonaceae, the onset of high diversification rates of Malmeoideae is speculated to be related to the Terminal Eocene Event (TEE, ca. 34 Ma), which involved a sharp drop in global temperature and likely promoted vicariance events (Xiang and Soltis, 2001; Xiang et al., 2018). The timing of diversification rate shifts in *Guatteria* and *Pseuduvaria* is instead aligned with the end of Mid-Miocene Climatic Optimum (MMCO, ca. 16–14 Ma): the resultant sudden global cooling and drought forced species to encounter new ecological opportunities for speciation. Similar patterns have been observed in other plant lineages, such as *Hypericum* (Hypericaceae), in which massive mountain formation and climate cooling following the last thermal maximum in the Miocene stimulated global radiation (Nürk et al., 2013).

### 4.2 Do diversification rates reflect climate niche lability?

The plot of niche evolution over time (**Figure 4**) reveals that the acceleration of climate niche (ca. 15 Ma) appears to be later than the rapid increase in the diversification rate of Annonaceae (ca. 25 Ma). The onset of species diversification around 25 Ma may have occurred in ancestral areas, where the “Late Oligocene Warming Event” (LOWE, ca. 26–24 Ma) provided optimal global climate conditions for tropical forest biome development, leading to expanded habitats that promoted species dispersal and exchange. The initial diversification may therefore reflect niche conservatism, as no rapid increase in niche evolutionary rate was observed before the mid-Miocene. Shifts in niche evolution appear to be associated with increasing seasonal environments (**Supplementary Table S7**; **Supplementary Figure S6**), which lag behind species diversification. It is noteworthy that *Asimina* is the only Annonaceae genus fully adapted to freezing temperatures (Hormaza, 2014), and it is a good example of a genus which experienced a climatic niche shift following migration from tropical East Asia to temperate North America (Kral, 1960; Li et al., 2017), and following the evolution of protogynous flowering with an extended flowering period (Kral, 1960; Willson and Schemske, 1980; Lagrange and Tramer, 1985; Norman and Clayton, 1986; Norman et al., 1992; Cox, 1998; Losada et al., 2017) and stigmatic secretions that cover all stigmas (Losada et al., 2017). The evolution of these traits allows *Asimina* to thrive in colder and drier habitats by increasing pollination rates during adverse environmental conditions (Saunders, 2020). A previous study of biotic correlations (Xue et al., 2020) also demonstrated that Annonaceae possess adaptive traits that allow them to thrive in diverse climatic conditions, indicating their potential for expansion beyond the tropics.

Ramirez-Barahona et al. (2020) observed a general pattern in angiosperms in which lineages tend to diversify shortly after acquiring the ability to occupy temperate and arid biomes. However, our study of Annonaceae reveals that its early diversification was driven by niche conservatism (**Figure 4**) and that the rapid increase in diversification rate lagged behind niche evolution. Interestingly, following the initial diversification, Annonaceae expanded into temperate and arid climates. This difference suggests that Annonaceae might have undergone its diversification primarily in tropical regions despite the presence of temperate-adapted lineages. This is uncommon but has also been documented in Saxifragales (Folk et al., 2019) and *Penstemon* (Plantaginaceae; Stone and Wolfe, 2021). Different timing patterns have been documented in other taxa. For example, in *Hypericum* (Hypericaceae) niche evolution precedes the burst in diversification rate, with the early adaptation to drought tolerance enabling them to thrive during the sharp global temperature drop following the mid-Pliocene thermal optimum (Nürk et al., 2013). In contrast to the interpretation by Folk et al. (2019) and Stone and Wolfe (2021), however, our results indicate that diversification in Annonaceae does not appear to be influenced by density-dependent factors i.e., niche spaces (Walker and Valentine, 1984; Nee et al., 1992). This is supported by the increasing trend in the net diversification rate (**Figure 4**). Our findings challenge the established concept of density-dependent diversification rate slowdown associated with the saturation of ecological niches (Morlon et al., 2010), but appears to be influenced by interspecific competition. The strong asymmetric floristic exchange from western to eastern Southeast Asia and from North/Central to South America observed in Annonaceae (**Figure 3**; **Supplementary Table S4**), suggests a link to interspecific competition, in which species might disperse from areas with higher to lower species richness. Western Southeast Asia and North/Central America, being old and species-rich regions, serve as common sources for dispersal (Bacon et al., 2015; Crayn et al., 2015; Dupin et al., 2017). Numerous studies have provided evidence that species dispersal enables them to evade competition, promoting opportunities to colonize new habitats (Van Den Elzen et al., 2016) and facilitating their adaptation to new environments (Economou, 1991; Martorell and Martínez-López, 2014; Burgess et al., 2016). This highlights the importance of geographical transitions in achieving niche evolution (Rosenzweig, 1995; Gómez-Rodríguez et al., 2015). Similarly, macroevolutionary studies suggest that following diversification, ancestral habitats became increasingly saturated by species accumulation, heightening pressure through interspecific competition. The availability of novel habitats, such as those outside the tropics, offered opportunities to escape intensified interspecific competition, resulting in an accelerated evolution of niches through adaptation to new environments (Aguilée et al., 2018; Folk et al., 2019).

Our findings also challenge the assumption that shifts in niche evolution directly dictate changes in diversification rate. Even though our QuaSSE model test results (**Supplementary Tables S10**, **S11**) suggest that the speciation rate is clearly correlated with PC raw values, the niche evolutionary test does not show a direct correspondence with shifts in divergence rate (**Figure 2**). A similar process might occur in other taxa: diversification in Terebinthaceae (Weeks et al., 2014) and phaseoloid legumes (Li et al., 2013) may not be driven by niche evolution as speculated, and instead possibly exhibit a similar diversification pattern as Annonaceae. Our study therefore provides a novel perspective for understanding diversification across disparate taxa.

### 4.3 Are particular biogeographical events associated with shifts in diversification rate in Annonaceae?

Compared with a large number of estimated biogeographical events, only two of five diversification rate shifts are directly associated with geographical events, indicating that geographical events do not always trigger diversification. Both mapped biogeographical events are vicariant, including *Guatteria*, which experienced rapid diversification in South America following vicariance. This finding is consistent with previous research by Erkens et al. (2007), which also supports the rapid diversification of *Guatteria* after its colonization of South America. The uplift of the Andes in South America, particularly during the Late Miocene and early Pliocene, created opportunities for diversification by promoting physiographic heterogeneity, regional climate diversity, parallel geographic speciation, and adaptive radiation (Garzione et al., 2008; Hoorn et al., 2010; 2013; Mulch et al., 2010; Luebert and Weigend, 2014; Lagomarsino et al., 2016; Mulch, 2016). Similar diversification patterns associated with Andean uplift have been observed in lineages of other plant families, such as the *Oxalis tuberosa* alliance (Oxalidaceae; Emshwiller and Doyle, 1998; 2002), *Lupinus* (Fabaceae; Hughes and Eastwood, 2006; Drummond et al., 2012), *Hypericum* (Hypericaceae; Nürk et al., 2013), and the Páramo clade in the Valerianaceae (Madriñán et al., 2013). Other intrinsic biotic factors of *Guatteria*, such as pollen monads and absence of anther septation, have been reported to be related to high net diversification rates (Xue et al., 2020). In contrast to *Guatteria*, the reasons behind the diversification rate shift in the other clades are more complex. The second branch with diversification rate shift in subfamily Malmeoideae (excluding tribe Piptostigmateae), comprises 45 genera and spans five biogeographical regions, excluding Africa. Recent global climate change and frequent tectonic movements may have contributed to diversification rate shifts in regions outside Africa (Maslin et al., 2005; Antonelli et al., 2018). However, the diversification of the two most species-rich subfamilies, Annonoideae and Malmeoideae, may be attributed to distinct evolutionary models. In the early originating subfamily Annonoideae, the diversification rate shift (N1) appears to be unrelated to geographical events, as vicariance events occur later than the diversification rate shift.

A similar pattern has also been reported in *Scleria* (Cyperaceae; Larridon et al., 2021), Adoxaceae and Valerianaceae (Moore and Donoghue, 2007). Those cases indicate that dispersal and diversification are not related in a simple deterministic way, suggesting the involvement of key innovations. The results of the GeoSSE shows that diversification was unrelated with species ranges in Annonaceae (**Supplementary Tables S5**, **S6**). Our findings overall do not provide conclusive evidence for a direct association between range evolution and species diversification in Annonaceae. It is important to note that not all shifts in diversification rate can be solely attributed to biogeographical events, as they need to occur in the right location and timeframe for their influence to be significant. The direction of asymmetric dispersal from areas with higher to lower species richness may imply the contribution of range evolution to the niche divergence resulting from diversification. These LDD events have been documented to be promoted by frugivores, including large-bodied mammals, as well as strong-flying birds or bats, enabling them to transport seeds across mountain or ocean barriers (Onstein et al., 2019; Lopes et al., 2023).

Comprehensive taxon sampling as well as estimation of biogeographical events in extinct lineages are required to further test hypotheses regarding the correlation between dispersification and biological characteristics, and the interplay between range and niche evolution in diversification. Our study further highlights the capacity of Annonaceae to disperse over long distances, enabling them to reach and establish in new geographic areas (**Figure 3**, **Supplementary Table S4**). Future research exploring the interplay between niche lability and range evolution will further enhance our understanding of the dynamics of tropical forest plant lineages.

### 4.4 Possible sources of uncertainty in the results

Our study is based on an extensive super-matrix comprising 835 taxa, representing the most comprehensive phylogenetic tree to date. We exclusively utilized chloroplast DNA regions to maximize species sampling, with the majority of the data obtained from herbarium-preserved leaves. The incorporation of nuclear gene fragments would significantly reduce the number of species in the phylogenetic tree, hindering our comprehensive exploration of species diversity. The uniparental inheritance of chloroplast regions can lead to limited genetic variation, however, potentially failing to capture the full evolutionary history of a species (Jansen and Ruhlman, 2012). Reliance solely on chloroplast genes for phylogenetic relationship reconstruction has been criticized for generally having lower mutation rates compared to nuclear genomes (Wolfe et al., 1987), resulting in reduced resolution in relationships among closely related species.

The unsampled species, representing 65.8% of recognized species, along with the uneven representation of species richness in certain clades, has the potential to introduce bias in estimating divergence time and assessing overall diversification (Gotelli and Colwell, 2001; Chao et al., 2005). This becomes especially critical when the missing entries in the data matrix are non-randomly distributed among taxa, as such discrepancies can interact with priors on topology and branch lengths, potentially leading to misleading results (Ronquist et al., 2012). Nevertheless, at the genus level, our sampling covers ca. 98% genera in the family. Given that 64.8% of phylogenetic relationships were well-supported, the impact of missing data may be minimal (Lemmon et al., 2009; Wiens et al., 2010).

The uncertainties associated with 35.2% of the branches in the phylogenetic tree nevertheless have the potential to yield inaccurate estimations of the timing and historical biogeographical events. This can consequently lead to misinterpretations of the correlations with key innovation and/or diversification shifts. Additionally, the absence of extinct lineages hampers the exploration of biogeographical events in deeper lineages, further impeding our ability to investigate the interplay between niche lability and range evolution. Inaccuracies in environmental data sources, along with sampling inaccuracies and biased distribution of occurrence data, can impact niche estimations. Incorrect locality data may result in erroneous inferences about species niches, and an inappropriate distribution density of records can introduce bias into the results (Peterson et al., 2008; Elith and Leathwick, 2009). Moreover, the SSE framework has faced criticism for having a high type I error rate in situations with an inadequate number of tips (Davis et al., 2013) and in the absence of state-dependent diversification (Rabosky and Goldberg, 2015). While acknowledging the inherent biases in the materials and methods we adopted, the results can still hold value with careful demonstration (Beaulieu and O’Meara, 2016; Helmstetter et al., 2023). These challenges are not unique to our study and are shared by many similar investigations (e.g., Weeks et al., 2014; Givnish et al., 2016; Xiang et al., 2016; Castro-Insua et al., 2018). Future efforts should consider incorporating fossil records and additional niche modeling methods to mitigate such biases.

## 5 Conclusions

Global temperature may provide a better explanation for acceleration in diversification than time and constant models in Annonaceae. Our findings contradict the hypothesis that niche evolution drives diversification of Annonaceae: species instead tend to escape interspecific competition after diversification, followed with later niche acceleration. There is no conclusive evidence supporting a direct association between range evolution and species diversification in Annonaceae. Shifts in niche evolution do not accord with shifts in diversification rate. Our findings contribute insights into understanding the past diversification patterns of tropical forest plant groups, with implications for assessing the future fate of tropical forests. We have yet to address the interplay among key opportunities, key innovations, and species diversification. A higher resolution phylogenetic tree with more complete taxon sampling and comprehensive analyses of range evolution are needed in future studies.

## Data availability statement

The original contributions presented in the study are included in the article/**Supplementary Material**, further inquiries can be directed to the corresponding authors.

## Author contributions

WL: Conceptualization, Data curation, Formal analysis, Writing – original draft, Writing – review & editing. RW: Formal analysis, Writing – review & editing. M-FL: Writing – review & editing. RF: Supervision, Writing – review & editing. BX: Conceptualization, Funding acquisition, Resources, Supervision, Writing – review & editing. RS: Conceptualization, Project administration, Resources, Supervision, Writing – review & editing.
