# Supplementary material for: Climatic and biogeographic processes underlying the diversification of the pantropical flowering plant family Annonaceae
Source: Front Plant Sci. 2024 Mar 8;15:1287171. doi: 10.3389/fpls.2024.1287171 (PMC10957689; doi:10.3389/fpls.2024.1287171)
Supplement: Supplementary file 1 [file DataSheet_1.pdf]

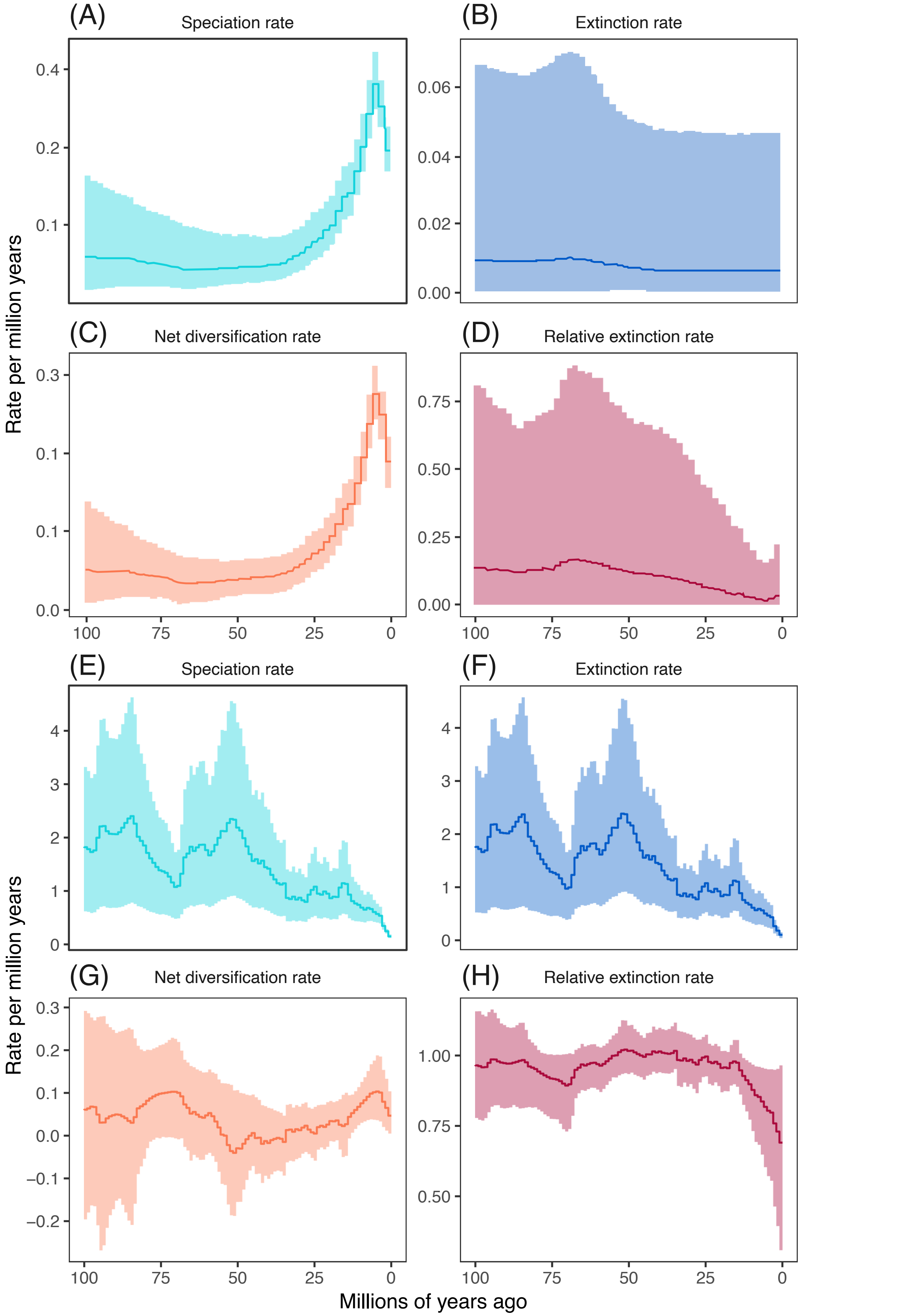


**Figure S1.** Summary figures of RevBayes estimations based on episodic birth-death model and environmental-dependent (paleotemperature) model for Annonaceae. (A) Speciation rate-through-time plot under episodic model; (B) Extinction rate-through-time plot under episodic model; (C) Net diversification rate-through-time plot under episodic model; (D) Relative extinction rate-through-time plot under episodic model; (E) Speciation rate-through-time plot as a function of temperature; (F) Extinction rate-through-time plot as a function of temperature; (G) Net diversification rate-through-time plot as a function of temperature; (H) Relative extinction rate-through-time plot as a function of temperature. The colored area representing the credibility interval, and the continuous line representing the median rate.
