# Supplementary material for: Climatic and biogeographic processes underlying the diversification of the pantropical flowering plant family Annonaceae
Source: Front Plant Sci. 2024 Mar 8;15:1287171. doi: 10.3389/fpls.2024.1287171 (PMC10957689; doi:10.3389/fpls.2024.1287171)
Supplement: Supplementary file 4 [file DataSheet_4.pdf]

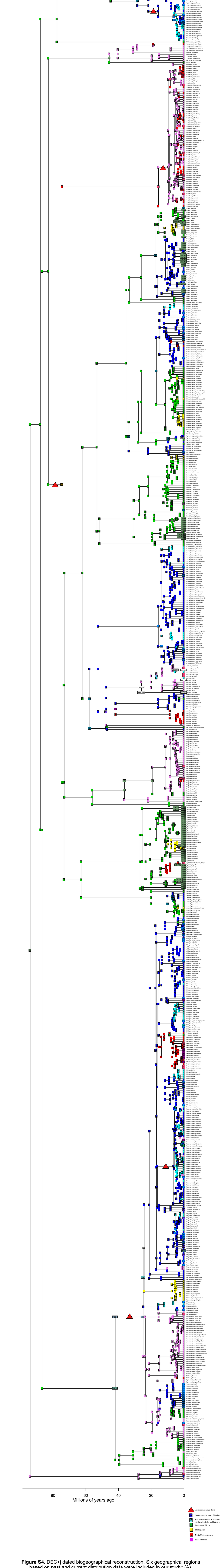

Figure S4. DEC+j dated biogeographical reconstruction. Six geographical regions based on past and current distribution data were included in our study: (A) Southeast Asia, west of Wallace's Line (dark blue); (B) Southeast Asia east of Wallace's Line, northern Australia, and Pacific islands (light blue); (C) Continental Africa (green); (D) Madagascar (yellow); (E) North/Central America (red); (F) South America (pink). Red rectangles represent diversification rate shifts.
