# Supplementary material for: Climatic and biogeographic processes underlying the diversification of the pantropical flowering plant family Annonaceae
Source: Front Plant Sci. 2024 Mar 8;15:1287171. doi: 10.3389/fpls.2024.1287171 (PMC10957689; doi:10.3389/fpls.2024.1287171)
Supplement: Supplementary file 5 [file DataSheet_5.pdf]

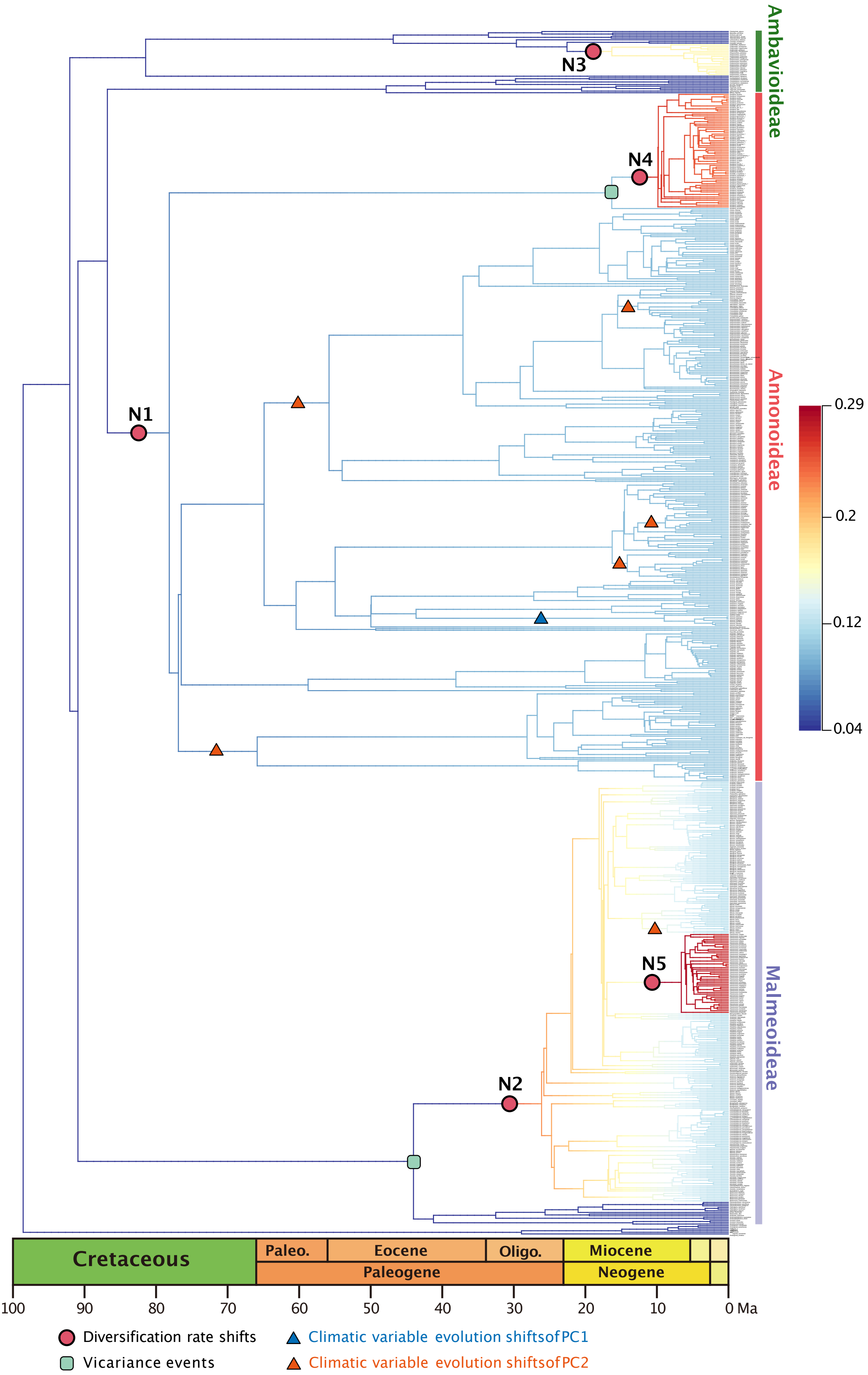

Figure S5. Best BAMM scenario, with diversification rate shift (shown in red dots) in Annonaceae, mapped positions of vicariance events (shown in the green square), climatic variable evolution shifts of PC1 (shown in blue triangle), climatic variable evolution shifts of PC2 (shown in red triangle). Branch colors indicating diversification rates.
