# Supplementary material for: Climatic and biogeographic processes underlying the diversification of the pantropical flowering plant family Annonaceae
Source: Front Plant Sci. 2024 Mar 8;15:1287171. doi: 10.3389/fpls.2024.1287171 (PMC10957689; doi:10.3389/fpls.2024.1287171)
Supplement: Supplementary file 2 [file DataSheet_2.pdf]

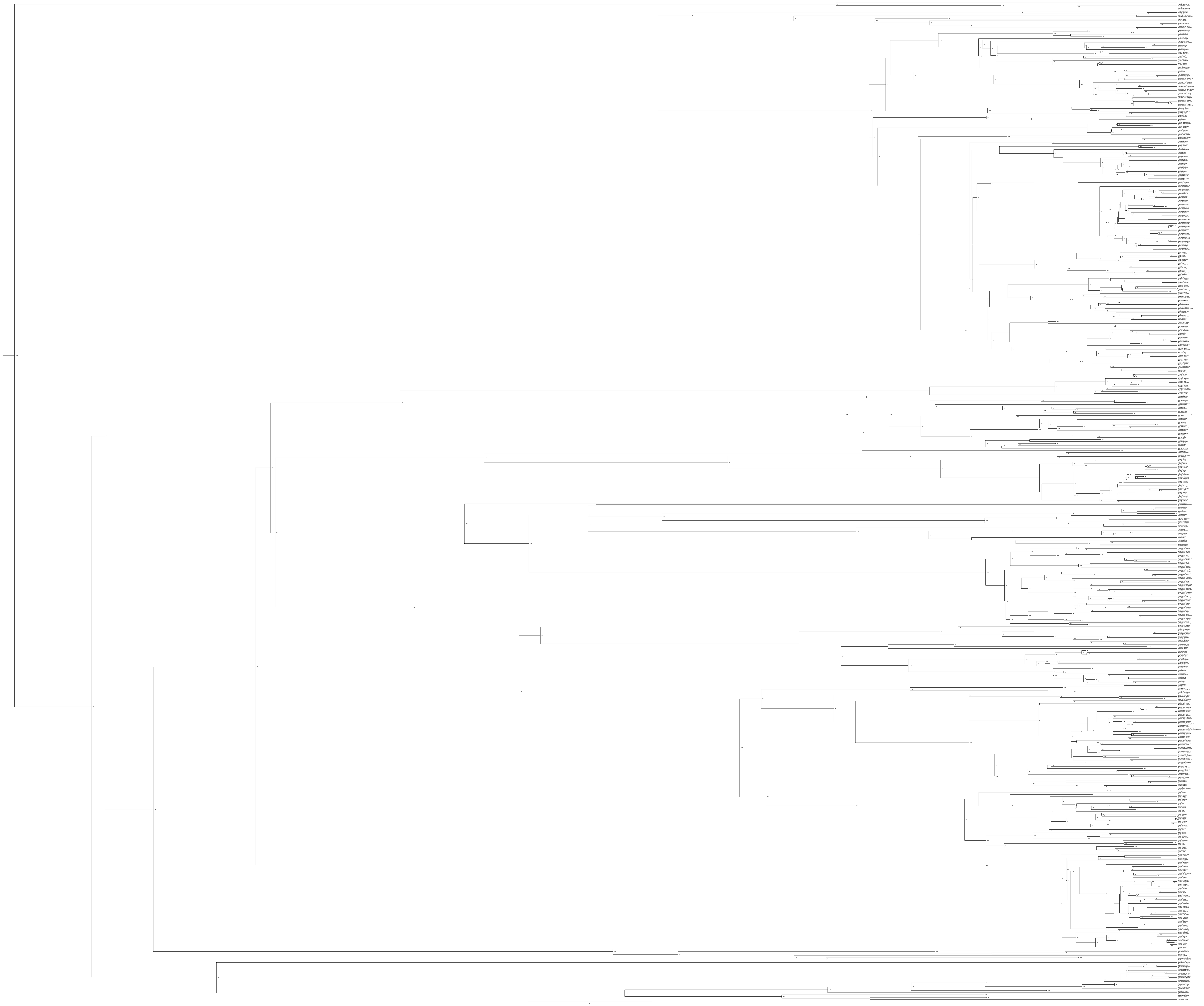

Figure S2. RaxML tree based on 728 taxa with supporting value at each node.
