# Supplementary material for: Climatic and biogeographic processes underlying the diversification of the pantropical flowering plant family Annonaceae
Source: Front Plant Sci. 2024 Mar 8;15:1287171. doi: 10.3389/fpls.2024.1287171 (PMC10957689; doi:10.3389/fpls.2024.1287171)
Supplement: Supplementary file 3 [file DataSheet_3.pdf]

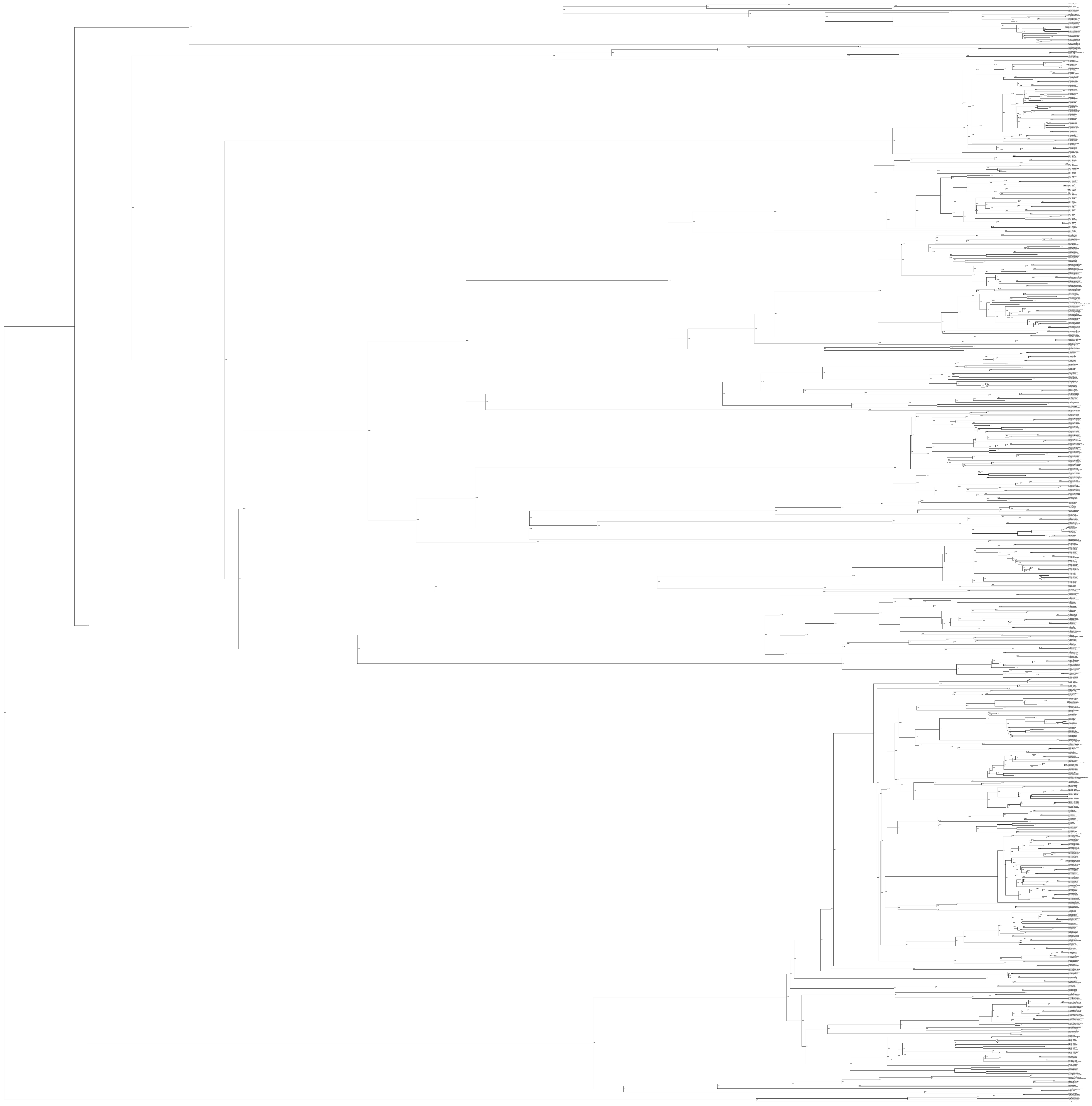

Figure S3. Number of internal nodes labeled on the phylogenetic tree based on the 835-taxon tree. (Divergence times should be checked in conjunction with Supplementary Table S2.)
