# Supplementary material for: Climatic and biogeographic processes underlying the diversification of the pantropical flowering plant family Annonaceae
Source: Front Plant Sci. 2024 Mar 8;15:1287171. doi: 10.3389/fpls.2024.1287171 (PMC10957689; doi:10.3389/fpls.2024.1287171)
Supplement: Supplementary file 6 [file DataSheet_6.pdf]

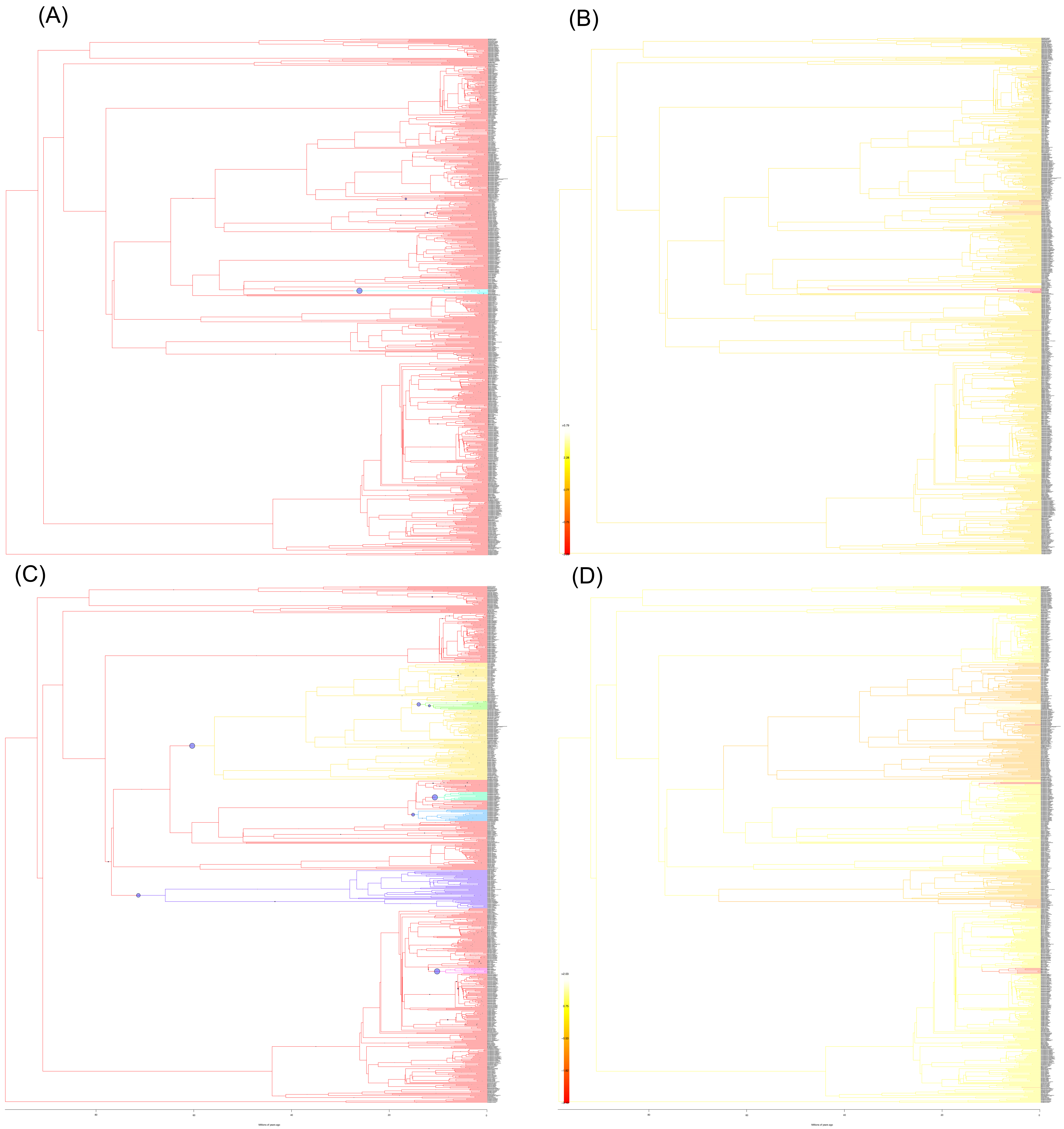

Figure S6. (A) Climatic variable evolution shifts of PC1; (B) Phenotype reconstruction for PC1; (C) Climatic variable evolution shifts of PC2; (D) Phenotype reconstruction for PC1. For (A) and (C), branch colors indicate niche evolutionary rate size, and optima shift (PP>0.5) are indicated with blue circles with size proportional to the posterior probability of each shift; For (B) and (D), branch color stands for phenotypic value according to the scale (bottom left).
